# Obesity, food habits and socio-demographic factors among university students in Germany: a cross-sectional study

**DOI:** 10.1038/s41598-026-57347-y

**Published:** 2026-06-14

**Authors:** Dominic Lemken, Ana Estevez Magnasco, Monika Hartmann, Leonie Bach, Antje Risius, Philipp Karl Seegers, Peter von Philipsborn

**Affiliations:** 1 Chair for Socioeconomics of Sustainable Nutrition, Institute for Food and Resource Economics, University of Bonn, 53115 Bonn, Germany; 2 Chair for Agriculture and Food Market Research, Institute for Food and Resource Economics, University of Bonn, 53115 Bonn, Germany; 3 Food and Consumer Sciences, Sustainable Nutrition and Distribution, Department of Nutritional Sciences, University of Applied Science Fulda, 36037 Fulda, Germany; 4 Economics of Education, School of Business and Economics, University of Maastricht, 6211 LK Maastricht, Netherlands; 5 Chair of Public Health Nutrition , Universität Bayreuth, 95447 Bayreuth, Germany

**Keywords:** Obesity, University students, Socio-cultural factors, Risk factors, Population health, Living conditions, Dietary patterns, Financial dependency, Risk factors, Ageing

## Abstract

Obesity represents a growing public health challenge globally, with rising prevalence among university students. Its causes extend beyond diet and exercise, encompassing socioeconomic background, and family education and migration history. This study examined obesity among German university students using data from the 2016 Studitemps survey, which included over 9,960 participants and mirrors key characteristics of national statistics and the largest social study of German students “Sozialerhebung 2016”. Logistic regression results indicate that with each additional year, the associated chance of obesity increases. Students whose parents hold university degrees had significantly lower associated chances of obesity—nearly half—compared to those whose parents had not attained any university entry-level qualification (OR = 0.536). Furthermore, living alone or with a partner, as opposed to in shared accommodation, was associated with higher odds of obesity (OR = 1.878), as was frequent use of food delivery services (OR = 1.498). Student income showed no association with obesity in either the regression or descriptive analyses; however, students with obesity appeared more reliant on student loans. Public and university initiatives should focus on at-risk groups by improving access to shared accommodations, promoting healthier food choices to reduce reliance on delivery services, and addressing cultural challenges faced by students from lower-education households. In conclusion, this study highlights the complex interplay of socio-demographic and lifestyle factors in obesity among university students.

**Supplementary Information:**

The online version contains supplementary material available at 10.1038/s41598-026-57347-y.

## Introduction

Non-communicable diseases (NCDs), including cardiovascular diseases, cancer, chronic respiratory diseases, and diabetes, are the leading cause of death worldwide, accounting for 71% of all deaths globally^1,2^. Key risk factors directly linked to the development of NCDs include overweight and obesity. Moreover, several risk factors contribute to the development of overweight and obesity, including unhealthy diets, low physical activity, and sedentary lifestyle behavior^1,3–5^. As a key protagonist of chronic disease, obesity is a significant public health concern globally, with its prevalence steadily increasing in most demographics in most countries for which data are available. This steady increase also affects university students.

University life is recognized as a critical phase where lifestyle habits formed can substantially influence long-term health outcomes. Many countries, including Germany, lack comprehensive analyses of this demographic. Detailed data on factors such as income status and parental education are often unavailable or insufficiently reported. The rising incidence of obesity even among university students is concerning, due to the associated risk of chronic diseases such as diabetes, cardiovascular disease, and certain cancers^6,7^. More specifically, several diseases related to obesity normally found in later stages of life, show a growing incidence among younger population groups. Such is the case with diet-related-NCD such as heart disease and early onset of type 2 diabetes^6^. More importantly, considering that the Germany has a big proportion of its population with different education levels, and family origin background, understanding how these determinants of health may impact their life is key to approach and develop and equitable policy development^8,9^. Policies that consider the reality and challenges of all and not only the traditionally well-educated, stereotypical national family, applicable specifically to the German and worldwide context.

As young adults, students consolidate habits they acquired during their childhood and adolescence and develop new ones that will be present during the rest of their lives, as it has been shown for food, alcohol and nicotine consumption^1,10,11^. Additionally, obesity can impair not only physical health but also academic performance and overall quality of life, leading to long-term socioeconomic consequences^12^. The transition to university life often involves changes in dietary habits regarding food preparation, physical activity levels, and stress management, all of which are crucial in impacting the risk of overweight and obesity^11,13^. University students are susceptible, for example, to limited disposable income, restricted cooking skills, and insufficient nutrition knowledge, leading to poor food choices^11^. Moreover, this period can exacerbate and expose students to pre-existing or new ranges of stressors due to their transition process from adolescence to adulthood, such as academic pressure, changes in peer and family social supports and the exposure to participating in risky behaviours such as alcohol and drug use^11,13^. The implications of obesity extend beyond immediate health concerns, potentially leading to chronic diseases that affect long-term health and economic productivity. The need for integrated socio-political as well as scientific approaches to improve dietary patterns at the population level is clear^14^.

Health equity is a critical lens through which the issue of obesity should increasingly be examined. This also applies to Germany. Even before student life, socio-economic disparities already exist in children, where obesity rates are 9.9% among lower and 2.3% among higher socio-economic status groups based on the KIGGS panel^15^. In 11 years the prevalence of overweight among children from lower socioeconomic households increased significantly (from 20% to 25.5%), while it decreased among children from higher socioeconomic status households (from 10.2 to 7.7%)^15,16^. These disparities continue into adolescence. For young adults, stress plays a crucial role in the development and progression of obesity. In Germany, chronic stress is particularly associated with lower socio-economic status^17^. Factors such as access to nutritious food, opportunities for physical activity, and health education can be limited for all population groups, including university students, potentially exacerbating the risk of obesity^18–20^. Additionally, cultural and familial influences, particularly among university students with a migration background, suggest a linked to obesity prevalence in some countries^21^. In Germany, official statistics on university students and obesity are lacking, so a representative assessment of obesity-related socioeconomic factors and dietary behaviors is still missing.

By analyzing these variables, this study takes a multifaceted approach that acknowledges the heterogeneity among university students. It aims to contribute to the development of targeted public health interventions and policies that promote health equity while effectively addressing obesity within this population. Given the complex interplay of socio-demographic and lifestyle factors, this study aims to address the following research questions in the German context:

1. Are socio-demographic factors (age, sex, education of parents, migration status and income) associated with obesity among university students?
2. Are lifestyle and dietary factors (living situation, relationship status, food preparation modes, food category consumption frequencies) associated with obesity among university students?

## Methods

### Sampling strategy

In the present analysis, we utilized data sourced from the Fachkraft 2030 survey (at the time Fachkraft 2020, just Fachkraft hereafter), conducted by Studitemps in collaboration with Maastricht University, School of Business and Economics (SBE), Department of Macro, International and Labour Economics (MILE) in 2017. The data was designed to assess the economic and general life circumstances of higher education students across Germany. The data includes a broad participant base through an invitation distributed by Studitemps via the Jobmensa network, a large job platform for students. The dataset for our study comes from the 10th wave of this biannual cross-sectional survey, which was conducted online from March 22 to April 24, 2017, using Survey-gizmo, now Alchemer, as the survey management tool. Since 2012, the survey has been conducted in 25 waves, amassing responses from more than 400,000 students up to the winter semester of 2024. The 10th wave is the first—and so far only—wave to include questions related to nutrition and physical health. As a result, earlier waves of the Fachkraft data have not been used in public health research. Previous applications have primarily focused on labor economics^22^. However, during the COVID-19 pandemic, the data also contributed to understanding students’ attitudes, emotional responses, and behavioral changes in response to the crisis, resulting in a publication aimed at a more medically oriented audience^23^. More detailed information on the data, how it was measured and the different survey questions are available online (https://jobvalley.com/pdf/2019_Studie_Fachkraft2030.pdf).

For this specific study, the initial dataset comprised raw data from 11,648 student respondents. Screening and exclusion criteria were applied to ensure the validity of the data. These criteria led to the exclusion of several subsets of respondents in the following order: 386 students (3%) were excluded for failing to report on weight or height; 11 students (0.1%) provided implausible responses regarding food preparation; 207 students (2%) were under the legal age for participation (< 18); 45 students (0.4%) reported an income exceeding €10,000 per income source, which was considered implausible; 141 students (1.2%) used less than one-third of the median response time (< 12 min), suggesting insufficient engagement with the survey; 599 students (5%) had recently completed university education; and 292 students (3%) were high school students anticipating university studies, thus not currently within the target demographic. After these exclusions, the refined dataset includes 9960 students in Germany.

### Representativeness of the sample

An assessment of the representativeness of the Fachkraft sample reveals important insights regarding its alignment with the broader German student population, as outlined by federal statistics^24^. Historically, the Fachkraft survey data shows little systematic differences in most demographic categories compared to federal statistics, which compile pooled administrative information from universities across Germany. However, an overrepresentation of women in the survey has been observed, a common phenomenon in survey research attributed to higher response rates among women (Table 1).

The representativeness of the sample is further illustrated with the distribution of participants by German regions (Table 1). Although the regions are generally well-represented, some deviations exist, particularly North Rhine-Westphalia is underrepresented (Table 1). The mean age of survey participants (23.4 years) is similar to the national median (no mean available), although not perfectly comparable. Additionally, there is a noted underrepresentation of students enrolled in dual-study programs by 3% percentage points, a unique aspect of the German education system (Table 1).

**Table 1:** Socio-demographics of the 19. Fachkraft wave (this study’s sample) and federal statistics on students in Germany.

		10. Fachkraft survey wave (2016/17)	Destatis 2016/17*
Age	Age [years] [median]*	23	23.6
Sex	Women	0.55	0.48
Higher education	Research University	0.58	0.63
	University of Applied Sciences	0.42	0.34
State of university	Baden-Württemberg	0.14	0.11
	Bavaria	0.14	0.15
	Berlin	0.08	0.07
	Brandenburg	0.02	0.02
	Bremen	0.02	0.01
	Hamburg	0.04	0.03
	Hesse	0.09	0.10
	Mecklenburg-Western Pomerania	0.01	0.02
	Lower Saxony	0.07	0.09
	North Rhine-Westphalia	0.23	0.31
	Rhineland-Palatinate	0.04	0.05
	Saarland	0.01	0.01
	Saxony	0.05	0.05
	Saxony-Anhalt	0.02	0.02
	Schleswig-Holstein	0.03	0.02
	Thuringia	0.02	0.02
University system	In person program	0.96	0.93
	online learning only	0.02	0.02
	dual private-public	0.02	0.05

*Destatis= German student population by federal statistics^24^. The destatis median is calculated on a monthly basis (median = 23.6), the 10th Fachkraft calculates age on a yearly basis (median = 23 years). The interquartile range (IQR) of age is 4 years. Other variables are presented as percentage values.

Further, comparative analysis with the “Sozialerhebung 2016”^25^, a comprehensive student survey conducted every four years by the German Centre for Higher Education Research and Science Studies alongside the Studierendenwerke, validates the representativeness of other observed variables. The Fachkraft sample mirrors the “Sozialerhebung” in terms of the proportion of employed students, which is particularly relevant given that participants are recruited through Jobmensa, a job network for students (Student job, Supplementary Material S1), as well as concerning companionship and income (Supplementary Material S1). The “Sozialerhebung 2016” features more individuals living alone or with their partner (Supplementary Material S1). The statistical power to analyze obesity is sufficient. Based on a regression-oriented power analysis that accounts for the number of variables in the model and assumes a partial correlation of 5%, the actual sample size of 9,960 individuals yields a statistical power of 1.

### Data analysis

For our data analysis we employed Stata as our primary analytical tool. We computed Body Mass Index (BMI) based on self-reported weight and height at separate intervals throughout the survey. Our aim was to provide descriptive statistics pertaining to obesity rates. Weight status was dichotomized into obese versus non-obese; overweight was not analyzed as a separate outcome. We conducted two primary types of comparisons. Firstly, we compared obesity rates, using graphical representations of mean values and 95% confidence intervals, enabling us to discern significant differences. The confidence intervals are, explicitly mentioned in the text in brackets. Secondly, we compare cohorts of students with and without obesity graphically using mean values of multiple characteristics. To ascertain statistical significance in these cases, we employed the Mann Whitney U-Test when comparing two groups and the Kruskal-Wallis Test when comparing three or more groups to address non-normal distributions. Lastly, we utilized logistic regressions to estimate the probabilities associated with various factors, addressing missing values through case-wise deletion. To account for potential causal ordering and mediation, we employed a sequential modeling approach. Model 1 established a sociodemographic baseline (age, sex, parental education, and income). Model 2 expanded this by adjusting for adult social context (living situation and relationship status). Finally, Model 3 incorporated potential behavioral correlates, specifically food preparation practices. This sequential structure allows for the observation of how structural factors are potentially mediated by more proximal lifestyle behaviors.

## Results

### Age and sex

**Fig. 1:**
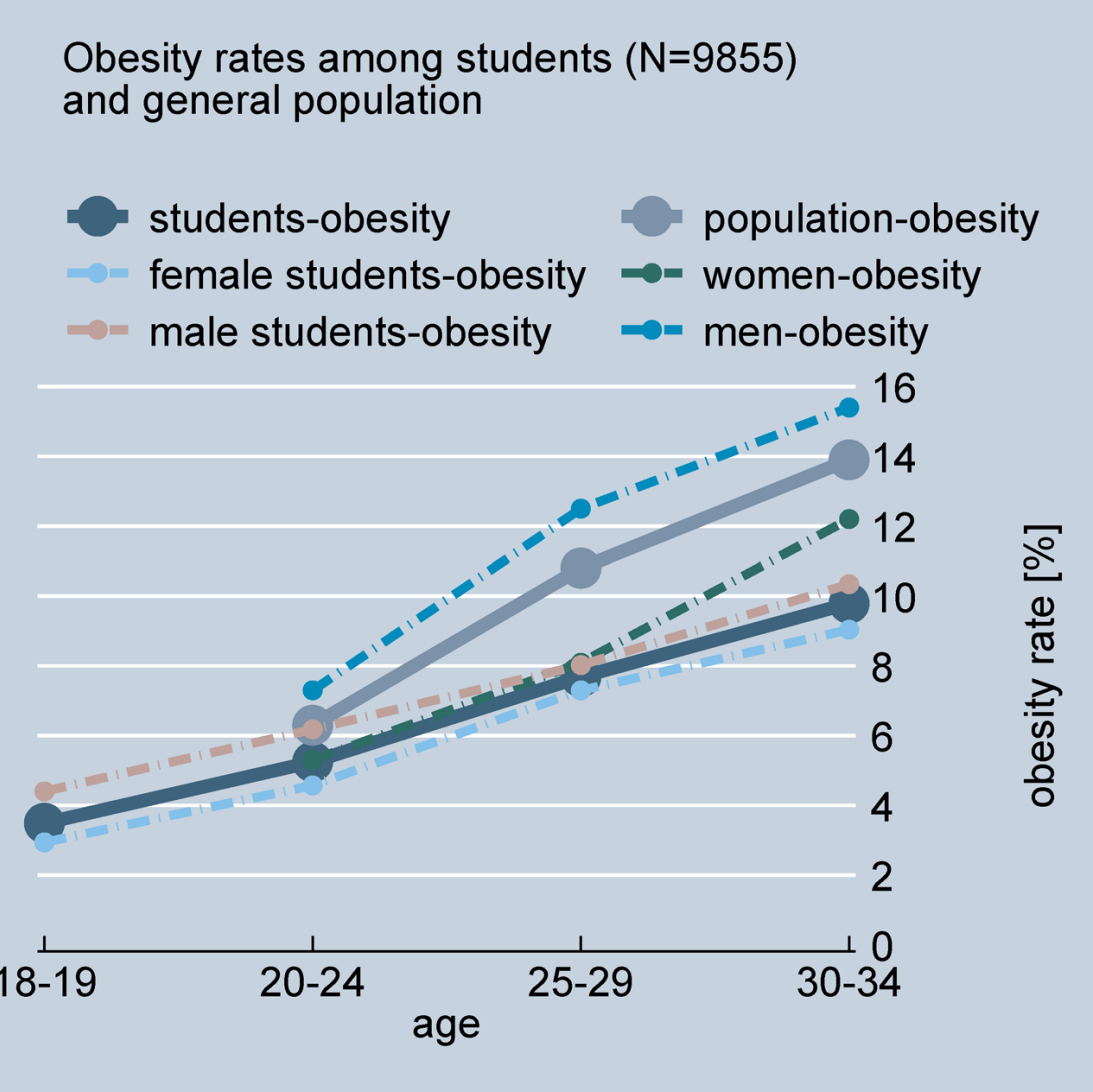
University students’ obesity rates in Germany. Obesity rates by age. Obesity rates among students are based on the sample (*N* = 9855), Obesity rates in the general population are based on the German Microcensus^23^.

Among students, obesity rates rise from 3.4% in the 18–19 age group to 9.8% in the 30 to 34 age group (Fig. 1). While both women and men experience similar trends in growth of obesity prevalence with age, women consistently exhibit lower rates across all age groups. Of note, differences between population^26^ and student obesity increase from 25 years onwards.

### Parents: education and migration

Two key formal educational qualifications are recognized in the following: firstly, the “(Fach)Abitur,” a secondary school diploma necessary for university entry level in Germany (UEL); and secondly, university degrees (UD) such as Bachelor or Diploma degrees, although the former is uncommon among the parent generation in Germany. The prevalence of UEL and UD among students’ parents can be accessed in the Supplementary Material S2.

**Fig. 2:**
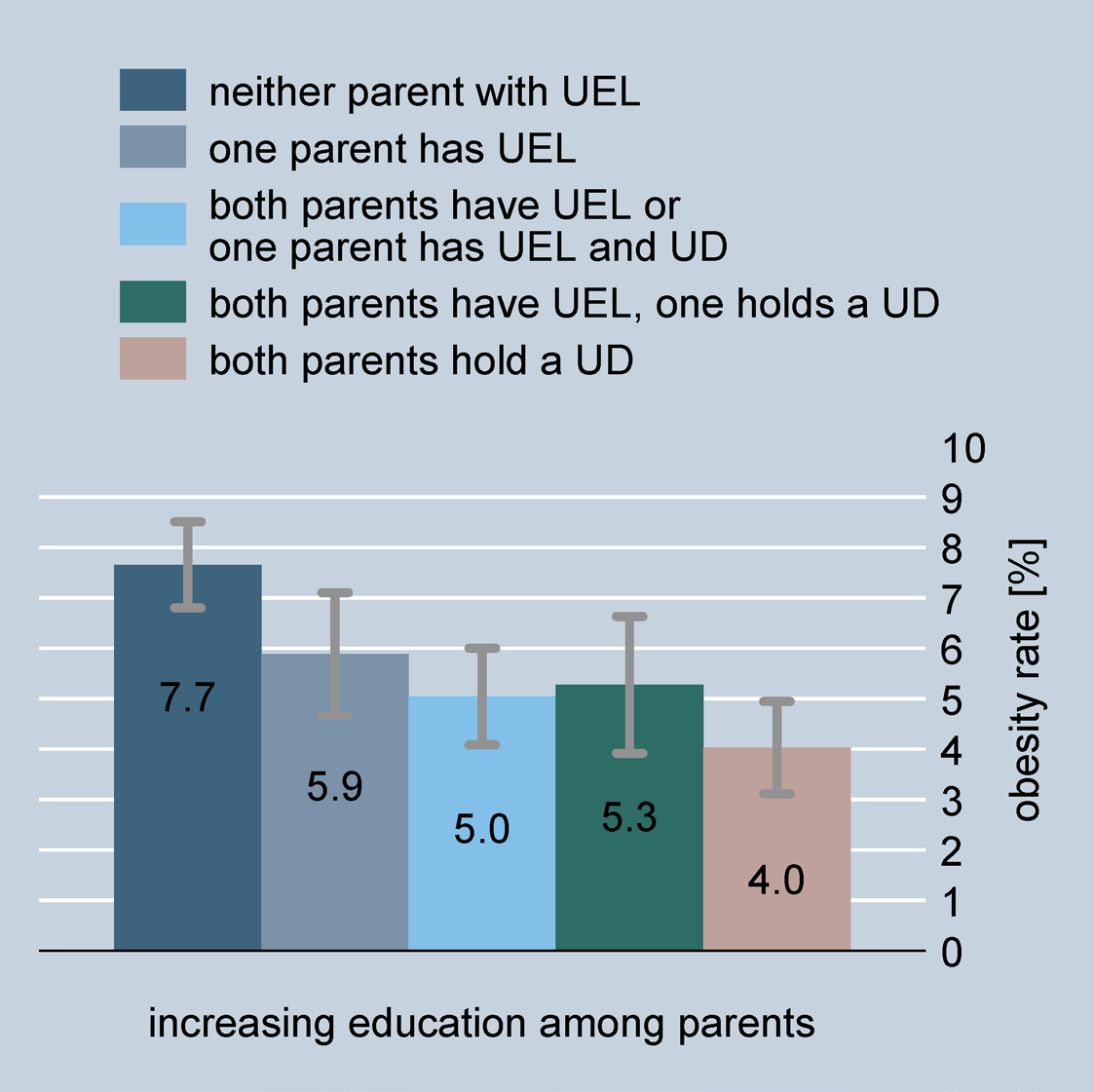
Education of parents by university entry level (UEL) and university degree (UD), (*N* = 9960)

We see a disparity in the educational backgrounds of parents of students with obesity compared to their peers (Fig. 2). The differences are particularly pronounced for the most and least educated groups. A university degree of both parents is linked to an obesity rate of 4% [3.1–4.9] among students, which is significantly lower than students with parents with none of these formal education achievements, where the rates are at 7.8% [6.8–8.5]. Even 1 parent with UEL is already a significant difference to none (*p* = 0.0272).

**Fig. 3:**
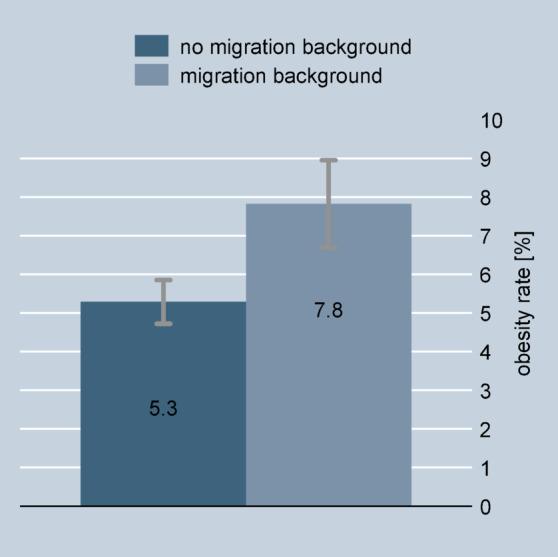
Obesity rates depending on migration status of at least 1 parent, (*N* = 8189)

The migration background of student families is diverse, commonly including origins such as Turkey, Russia, Poland, and Vietnam. Although only 82% of students disclosed their migration background, a group comparison is still valid. Here, students with and without migration background show a significant difference (*p* = 0.0001) in obesity rates, at 5% [4.7–5.8] and 8% [6.7-9.0] respectively (Fig. 3).

There are large differences between migration origins, e.g. 12% for Turkish immigrants and 5% for Polish ones, but limited subgroup size (372 and 137 respectively) does not allow for reliable estimates.

### Student income

**Fig. 4:**
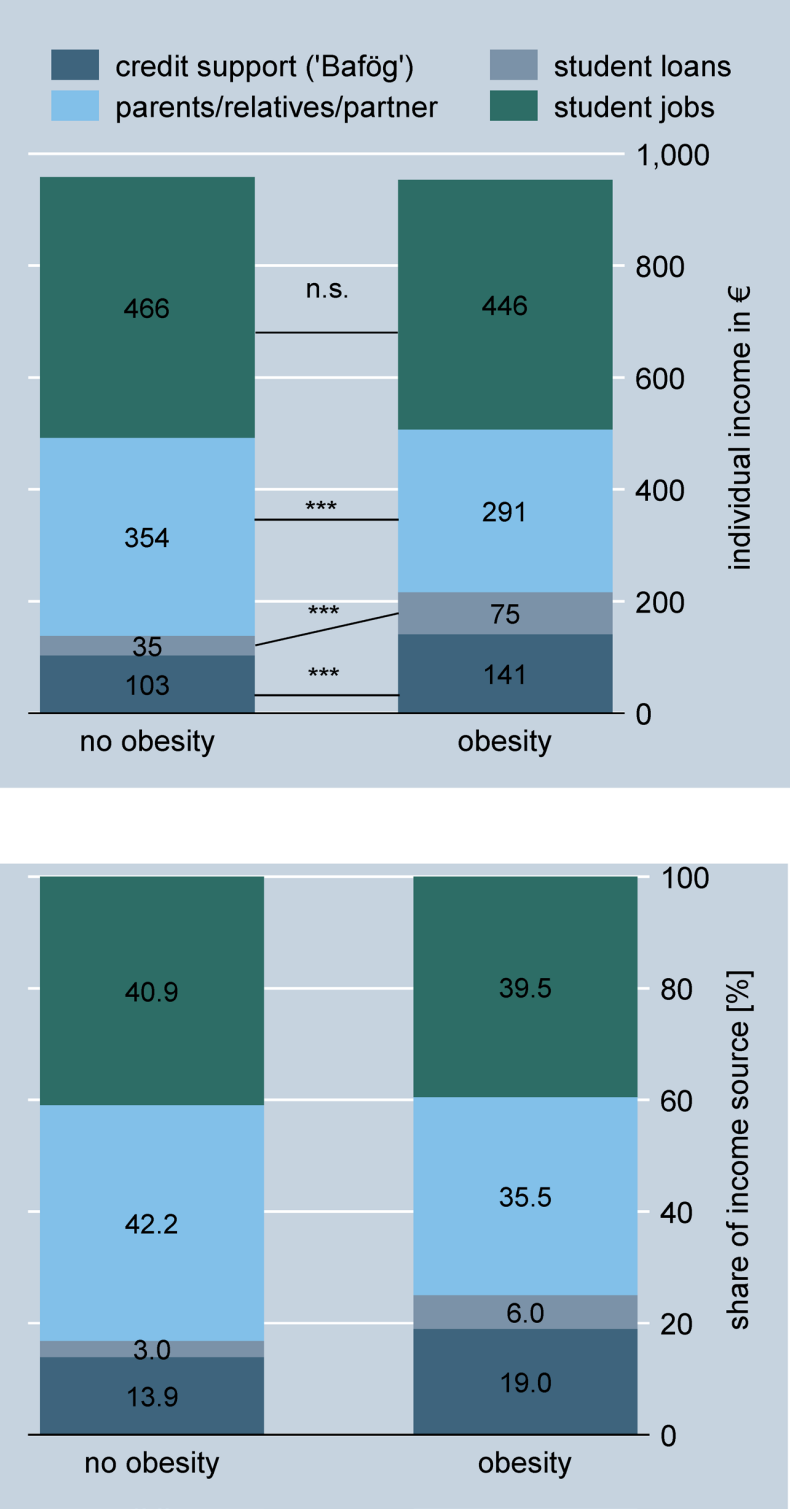
Average income and income source between students with and without obesity [in €], The figure compares overall income between students with and without obesity. Additionally, it compares the 4 main income sources between the 2 groups. *N* = 9959, n.s.=non significant, ***highly significant with *p* < 0.01.

In Germany, students rely on four primary sources of income: (a) the subsidized public student loan system (Bafög), (b) private sector student loans, (c) income from private individuals such as parents, relatives, friends, or partners, and (d) the student job market, which encompasses all types of formalized part-time employment. Overall, there is no significant difference in total income (*p*=0.5689) between students with obesity, who average 953€ [878-1029], and students without obesity, who average 958€ [939-977]. However, significant differences exist in the sources of income, with students with obesity receiving more from loans with 216€ [163-268] vs. 138€ [129-146€] (*p*=0.000) and students without obesity obtaining more from private individuals with 354€ [305-329] vs. 291€ [194-281] (*p*=0.000) (Figure 4). Income from student jobs is similar, although students with obesity exhibit a 5% points lower employment rate (p-value= 0.0235).

### Relationship status and living situation of students

**Fig. 5:**
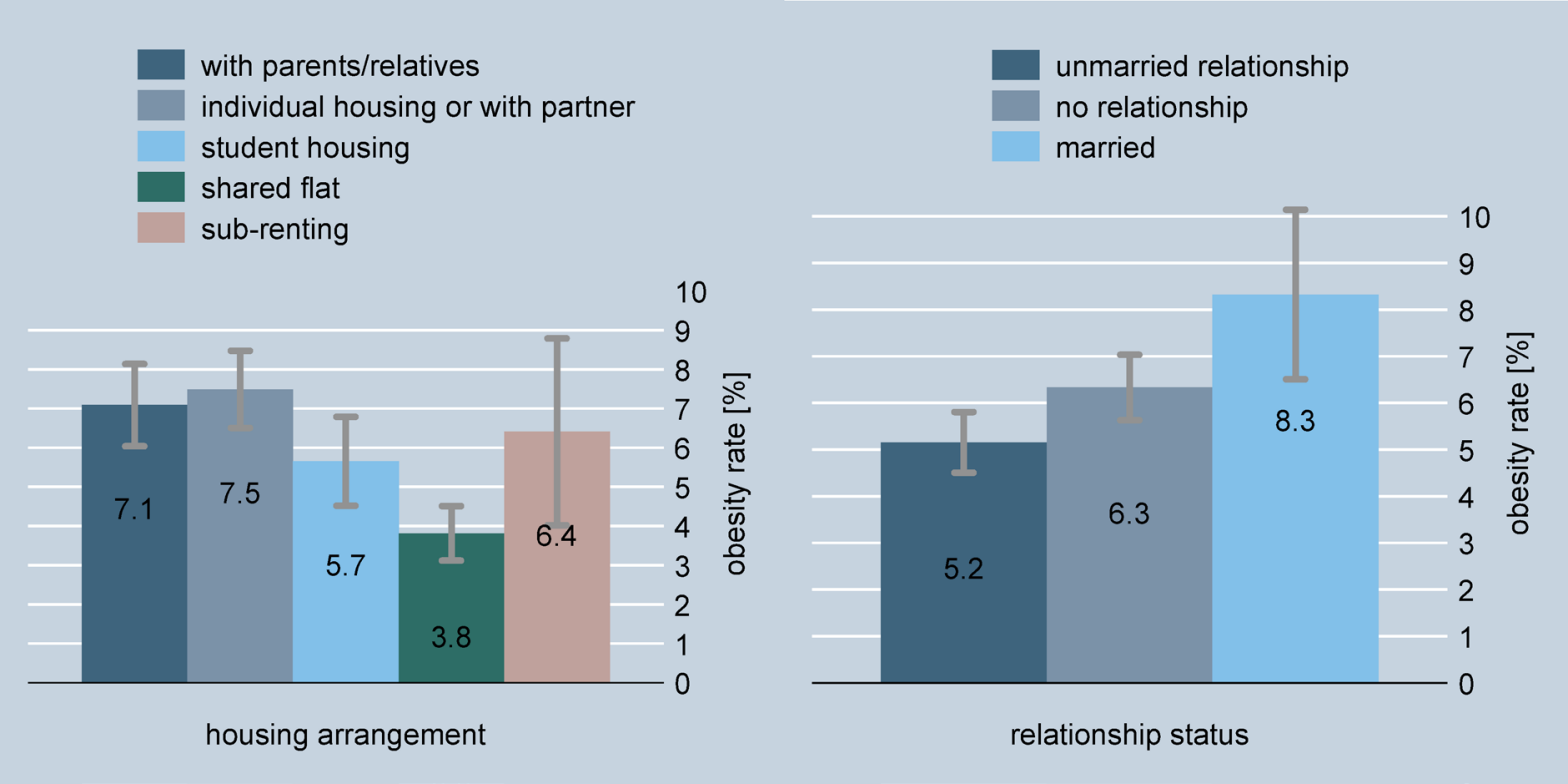
Obesity rates by living situation (*N* = 9960) and by relationship status (*N* = 9960).

The primary forms of student living arrangements include residing: (a) with parents or relatives, (b) alone or with a partner, typically a joint household arrangement, (c) in student-specific housing, (d) in a shared flat, typically consisting of joint and individual household structures, e.g. through sub-renting. Sub-renting is the least common, with only 4% of students currently engaged in such arrangements. This scarcity leads to great uncertainty in estimating obesity rates for this group. Obesity rates are lowest among students living in shared flats, at 3.8% [3.1–4.5], whereas the highest rates, ranging from 7.1% [6.0-8.1] to 7.5% [6.5–8.5], occur among students living with parents, relatives, alone, or with a partner (Fig. 5). Here, the obesity rate among students living in student housing differs significantly from those living alone or with a partner (*p* = 0.0208), but not from those living with parents or relatives (*p* = 0.0745).

Additionally, significant differences in obesity rates correlate with relationship status; unmarried students in relationships exhibit the lowest rates with 5.2% [4.5–5.8], while married students show a significantly higher rate at 8.3% [6.5–10.1] (*p* = 0.0002) (Fig. 5). Married students are also the oldest group with an average age of 24.4 years compared to 22.9 and 23.4 for no relationship and unmarried relationship. 2.3% of all students have children; 12% [7.8–16.4] of students with children have obesity, but the small number of cases precludes a reliable estimate.

### Food habits of students

**Fig. 6:**
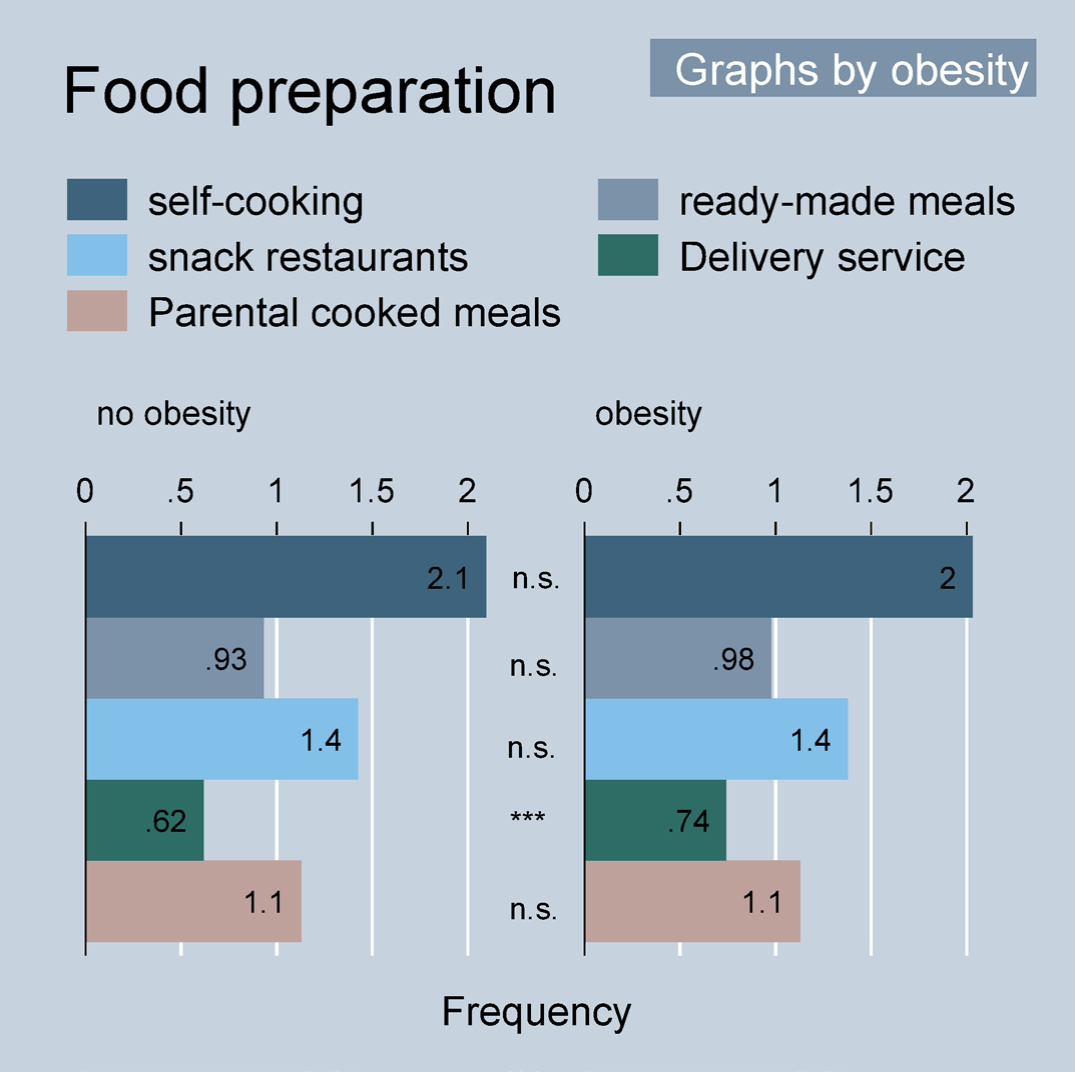
Food preparation habits between students with obesity and non-obese students (*N* = 9960). Likert Scale, ranging from 0(never),-1(once per week or less), 2(multiple times per week) to 3(daily), n.s.=non-significant, ***=highly significant with *p* < 0.01.

We examine how often students engage in cooking for themselves, dining at canteens and restaurants, consuming meals prepared by parents or relatives, consuming ready-made meals, and ordering from food delivery services. Inquiring about the frequency with which students prepare or obtain food through these channels—ranging from never to once per week or less, multiple times a week, to daily—reveals that students with and without obesity have on average similar habits, with the exception of food delivery services. Specifically, a Mann-Whitney U Test indicates no significant differences in the use of ready-made meals (*p*= 0.1427) or any other food acquisition methods. However, students with obesity significantly order more often from delivery services (*p*=0.0001) with a frequency score of 0.74[0.70-0.79] vs. 0.62 [0-61-0.63] (Figure 6).

**Fig. 7:**
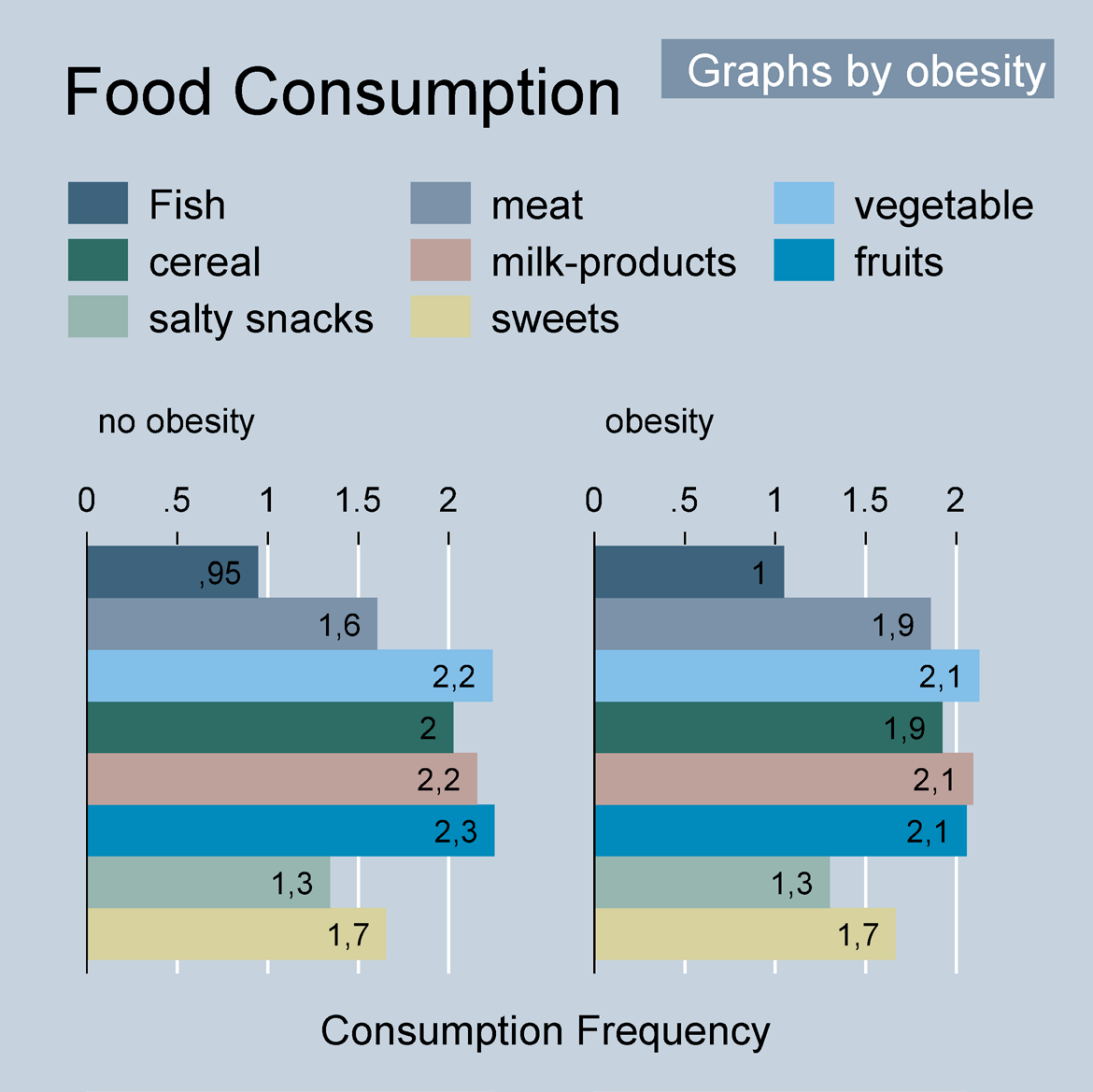
Food consumption frequencies of major food groups (*N* = 9960). Consumption scale ranging from 0(never),-1(once per week or less), 2 (multiple times per week) to 3 (daily).

Similarly. the frequency of consumption across various food categories is largely comparable between students with and without obesity. The sole significant difference is in meat consumption, (*p* = 0.0001), where there is a large variance, with students with obesity averaging 1.9 [1.79–1.93] compared to 1.6 [1.58–1.62] for students without obesity on a 4-point consumption scale, (Fig. 7).

**Fig. 8:**
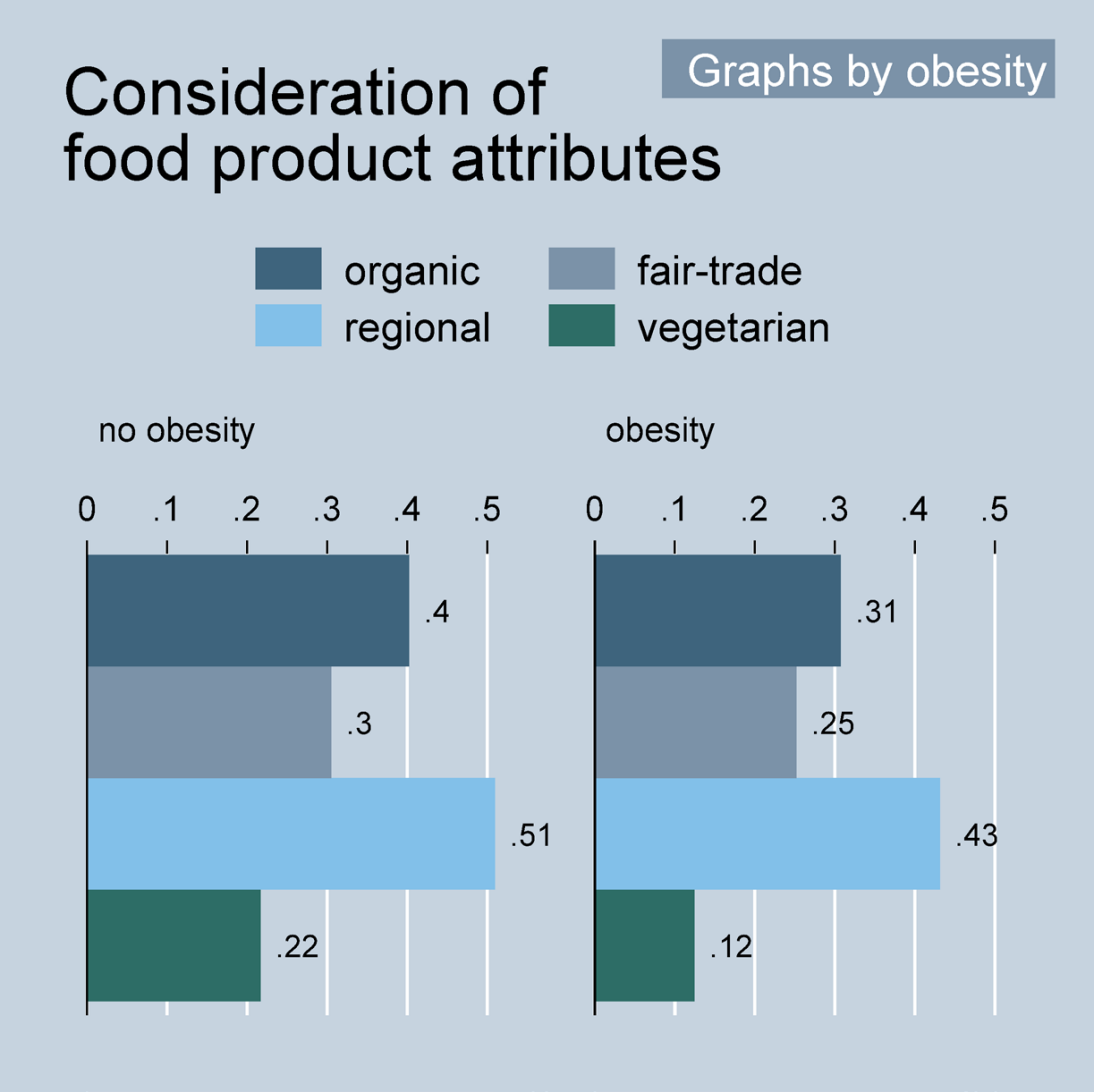
Consideration of food attributes (*N* = 9960). Question: “Do you value the following product attribute while shopping” − 0 (no) and 1 (yes).

Consumer interest in food product attributes differs significantly between attributes commonly labelled, namely organic, fair-trade, regional and vegetarian products. When asked “do you value the following product attribute while shopping”, students with obesity are less likely to consider all (*p* < 0.01). The implications of such consumer behavior are not clear at this point (Fig. 8).

### Odds of obesity in a multivariate analysis

**Table 2:** Odds ratios of obesity by socio-demographic characteristics across the total sample (10. Fachkraft Survey Wave 2016/17).

Explanatory variables	Odds-ratios	Ci95	*p*-value
Age [years]	1.208	[0.978,1.492]	0.080
Age^2^ [years^2^]	0.998	[0.994,1.002]	0.305
Gender [1=women, 0=men]	0.800	[0.676,0.947]	0.010
Reference category for parents’ education= neither parent has UEL.			
1=one parent with UEL	0.789	[0.612,1.017]	0.067
2=both parents have UEL or one parent has UEL and UD	0.672	[0.531,0.850]	0.001
3=both parents have UEL, one holds UD	0.717	[0.532,0.967]	0.029
4=both parents hold UD	0.536	[0.409,0.703]	0.000
Income [100€]	0.991	[0.974,1.007]	0.273
Income^2^ [100^2^ €]	1.000	[1.000,1.000]	0.436
Observations	9906		

Squared effects included for continuous variables. The brackets present the measurement of variables. Education of parents is always compared to the reference category of neither parent has UEL. Odds ratios present the Exponentiated coefficients; For significance assessments, P-values are displayed, 95% confidence intervals are displayed in the third column.

**Table 3:** Odds ratios of obesity by living situation and relationship status across the total sample (10. Fachkraft Survey Wave 2016/17).

Explanatory variables	Odds-ratios	Ci95	*p*-value
Age [years]	1.318	[1.063,1.635]	0.012
Age^2^ [years^2^]	0.996	[0.992,1.000]	0.069
Gender [1 = women, 0 = men]	0.828	[0.698,0.983]	0.031
Reference category for parents’ education= neither parent has UEL			
1 = one parent with UEL	0.809	[0.627,1.043]	0.102
2 = both parents have UEL or one parent has UEL and UD	0.701	[0.553,0.888]	0.003
3 = both parents have UEL, one holds UD	0.748	[0.554,1.009]	0.058
4 = both parents hold UD	0.578	[0.440,0.760]	0.000
Income [100€]	1.000	[1.000,1.000]	0.516
Income^2^ [100^2^ €]	1.000	[1.000,1.000]	0.709
Living situation - reference category in shared flat			
With parents or relatives	1.944	[1.504,2.514]	0.000
Alone or with partner	1.878	[1.471,2.397]	0.000
Student housing	1.416	[1.060,1.893]	0.019
Sub renting	1.514	[0.970,2.363]	0.068
Relationship status – reference category unmarried relationship			
No relationship	1.332	[1.107,1.603]	0.002
Married	1.466	[1.105,1.944]	0.008
Observations	9906		

Living situation is always compared to the reference category living in a shared flat, Relationship status is always compared to being in an unmarried relationship. For significance assessments, P-values are displayed, 95% confidence intervals are displayed in the third column.

**Table 4:** Odds ratios of obesity by food preparation mode across the total sample (10. Fachkraft Survey Wave 2016/17).

Explanatory variables	Odds-ratios	Ci95	*p*-value
Age [years]	1.298	[1.045,1.612]	0.018
Age^2^ [years^2^]	0.996	[0.992,1.001]	0.092
Gender [1 = women, 0 = men]	0.845	[0.709,1.008]	0.062
Reference category for parents’ education= neither parent has UEL			
1 = one parent with UEL	0.805	[0.624,1.039]	0.096
2 = both parents have UEL or one parent has UEL and UD	0.700	[0.552,0.887]	0.003
3 = both parents have UEL, one holds UD	0.746	[0.552,1.009]	0.057
4 = both parents hold UD	0.587	[0.446,0.773]	0.000
Income [100€]	1.000	[1.000,1.000]	0.390
Income^2^ [100^2^ €]	1.000	[1.000,1.000]	0.594
Living situation - reference category in shared flat			
With parents or relatives	2.086	[1.578,2.757]	0.000
Alone or with partner	1.826	[1.429,2.334]	0.000
Student housing	1.474	[1.102,1.973]	0.009
Sub renting	1.576	[1.009,2.463]	0.046
Relationship status – reference category unmarried relationship			
No relationship	1.364	[1.131,1.645]	0.001
Married	1.542	[1.161,2.049]	0.003
Food preparation mode [FFQ: 4 point Likert scale]			
Self-cooking	0.962	[0.849,1.090]	0.542
Ready-made meals	1.015	[0.892,1.155]	0.819
Canteen, restaurants	0.889	[0.776,1.017]	0.086
Food delivery services	1.498	[1.280,1.754]	0.000
Parental cooked meals	0.902	[0.813,1.000]	0.049
Observations	9906		

For Food preparation mode, the FFQ 4 point Likert scale was measured: 0 = never, 1 = once a week or less, 2 = multiple times a week, 3 = daily. For significance assessments, P-values are displayed, 95% confidence intervals are displayed in the third column.

We present odds ratios from three logistic regression models examining associations between a range of individual characteristics and the odds of obesity (Tables 2, 3 and 4). These multivariate models include variables simultaneously, allowing us to assess whether each factor remains associated with obesity when accounting for others. The results are presented sequentially. The initial model regresses obesity upon and sociodemographic (Table 2) factors. The next model specification adds living situation and relationship status to the explanatory variables (Table 3). Finally, food preparation mode is added to the previous model specification (Table 4).

In our study, we found that older age tends to associated with increased odds of obesity (OR = 1.208, *p* = 0.080), although this association is only marginally significant when controlling for other variables (Table 2). Additionally, women show lower odds of obesity compared to men (OR = 0.800, *p* = 0.010). Parental education is inversely associated with the odds of obesity. Compared to students whose parents have no UEL, those with one parent with UEL have slightly lower odds (OR = 0.789, *p* = 0.067), while those with both parents holding university degrees show substantially lower odds (OR = 0.536, *p* < 0.001). Furthermore, household income and its squared term are not significantly associated with obesity in this model (OR = 0.991, *p* = 0.273; squared term OR = 1.000, *p* = 0.436). However, living situation shows strong associations, where, compared to students living in shared flats, those living with parents or relatives have almost double the odds of obesity (OR = 1.994, *p* < 0.001) (Table 3). Similarly, students living alone or with a partner (OR = 1.878, *p* < 0.001), in student housing (OR = 1.416, *p* = 0.019), or in sub-renting arrangements (OR = 1.514, *p* = 0.068) also show elevated odds. Relationship status is also associated with obesity. Compared to students in an unmarried relationship, those without a partner have higher odds of obesity (OR = 1.332, *p* = 0.002), as do those who are married (OR = 1.466, *p* < 0.008). Moreover, food sourcing behavior shows varying associations. Compared to those who rarely use a given method, frequent reliance on food delivery services is associated with higher odds of obesity (OR = 1.498, *p* < 0.001) (Table 4). Meals prepared by parents are associated with slightly lower odds (OR = 0.902, *p* = 0.049), while other sources, such as self-cooking, ready-made meals, and canteen or restaurant meals, do not show statistically significant associations.

All independent variables are non-standardized, meaning the magnitude of odds ratios should be interpreted cautiously and not compared across variables. For example, each additional year of age is associated with 1.208 times the odds of obesity.

## Discussion

This study aimed to investigate the multifaceted nature of obesity among university students in Germany, with a particular focus on sociodemographic, lifestyle, and dietary factors. The findings provide critical insights into the underlying factors associated with obesity in this demographic, and those not associated. Such factors can be used to identify groups at higher risk for obesity, which may help to inform health promotion efforts. Obesity remains less prevalent among university students in Germany than in the general population; however, the likelihood of becoming obese increases steadily with age, both among students and within the broader population, with a marked rise after 25 years of age. Students whose parents both held university degrees had a lower chance of having obesity compared to those whose parents did not hold a university-entry-level degree. Moreover, when comparing living alone or with a partner with living in shared accommodation, we found higher odds of obesity, as well as more frequent use of food delivery services among students with obesity. Interestingly, from a socioeconomic perspective, although we did not find an income association, students with obesity seem to be more dependent on loans.

### Interpretation

The finding that older age is associated with increased odds of obesity is often corroborated, for example Szemik et al. observed an increase in BMI as study years progress^27^. They also found that this increase was explained by dissatisfaction with students own health status, financial deficiencies, and a high meat diet. Descriptively, this study also points to a higher meat-eating frequency in students with obesity. Unlike previous research, we do not confirm a positive association between lower household income and obesity^16^. However, in this study, this risk manifests specifically among students more reliant on loans. This loan dependency could contribute to elevated stress levels due to financial pressures, as students face the dual burden of repaying loans while aiming to complete their studies within a limited timeframe. Heightened stress from financial constraints may, therefore, exacerbate obesity risk, aligning with findings that stress can impact eating habits and weight gain^28^.

Parental education level also appears to play a key role, consistent with previous research linking parental education to BMI^29^. This relationship underscores the lasting influence of early-life socioeconomic conditions on health outcomes, even within a relatively young and highly educated population. In our data, individuals with the highest parental education had almost half the odds of being obese compared to those with the lowest parental education. This pattern suggests that the formative effects of parental education, shaping health behaviors, lifestyle norms, and access to resources during adolescence, continue to exert influence well into adulthood. Additionally, the migration background of students seems to be linked with higher obesity rates, supporting evidence from previous research that students with migrant parents are at increased risk^21^. This relationship could reflect various influences, including cultural dietary patterns, socioeconomic challenges that migrant families may face, and possibly limited access to health-promoting resources in neighborhoods with higher migrant populations. The level of education, as well as migration background, could both be related to a lack of family resources in terms of knowledge and the ability to provide healthy foods at home^30^. Such factors may create environments where maintaining a healthy lifestyle is more challenging for students with a migration background, although there are substantial differences depending on the migrant community at hand.

Focusing on the living situation shows strong associations with obesity. In our study, we saw that students living alone had almost double the odds of obesity compared to those in shared flats. This supports the proposed literature, where shared living environments may encourage healthier eating habits^31^. Nevertheless, it is worth noting that we speculate that students with obesity may find shared living arrangements uncomfortable, potentially due to fear of discrimination or stigmatization, making shared accommodation less desirable. Some of the students are still considered to be older adolescents or young adults, where research has found a relationship between depression, obesity, and poor dietary choices^10,32^. Additionally, students living alone may rely more heavily on food delivery services. As our regression model shows, reliance on food delivery services is significantly associated with higher odds of obesity, and such services have been repeatedly linked with a tendency toward less healthy meals^34, 35^. This increased reliance on delivery services among students with obesity further highlights the impact of living arrangements can have on diet and underscores the importance of supportive, social living environments in promoting healthier food choices. Such a pattern is not as prevalent among shared flats, where communal meal planning and preparation could be more common^33^.

Multiple risk factors correlate with obesity and contribute to the emerging obesity pandemic, highlighting the complexity and significance of the matter^14,36^. Therefore, several of those enumerated could potentially be reduced with preventive measures. Examples include the development of preventive educational interventions before students leave high school and transition into the university environment^37^. Additionally, the key role universities play in ensuring healthier food environments and supporting healthier habits should not be underestimated. These institutional settings can be crucial in developing informative sessions, educating students about the importance of good nutrition, and providing healthy food options on campus^11^. Where consequently, such eating habit formation can later have an enormous impact on the choices made in later years^37^.

### Strength and limitations

Our study possesses several relevant strengths. To our knowledge, it is the only study to provide detailed data on obesity prevalence and relevant determinants among university students in Germany. The sample is large and representative of the German university student population concerning state of residence, employment status, income, type of institution, and term of enrollment, enhancing generalizability. Data collection was supported by multiple quality assurance measures, contributing to the robustness of findings. This study also has several limitations. First, it relies on self-reported data for weight and height, which may introduce reporting biases. Second, we use BMI as the primary criterion for defining obesity, despite recognized limitations in its ability to reflect overall health accurately. Accordingly, we used the cutoff of 30 kg/m^2^ to classify students with obesity and to approach correlations with that measurement. Therefore, we cannot exclude that some individuals may have a larger BMI due to a higher lean-mass proportion, for example. Finally, the study’s cross-sectional design restricts our capacity to infer causality between the identified determinants and obesity, limiting conclusions to associations rather than cause-and-effect relationships.

### Implications for policy and practice

Transition phases (i.e. phases in life in which individuals are adapting to fundamental changes) are considered important for habit formation^38^. Leaving school and entering higher education is a critical transition phase for most students^20^. Though this study did not find search for correlations, the findings offer valuable insights for policies aiming to reduce obesity among university students. The observed increase in rates of obesity with age, migration background or students from lower educational backgrounds, underscores the need for targeted health interventions which could help improve odds. Health promotion programs on campuses could specifically focus on these at-risk groups. The observation that students with obesity are less likely to live in shared flats suggests that promoting affordable and accessible shared living spaces could potentially help support healthier lifestyles. On that note, policies that improve student access to community or shared accommodations might theoretically encourage positive health behaviors by fostering social support networks and healthier eating patterns within communal settings. Furthermore, since students with obesity demonstrate a higher dependency on loans, policy efforts could explore financial support and debt-relief programs to alleviate economic pressures for groups with severe health conditions. Some ideas around promotive health programs could be developed within university settings perhaps encourage healthier food behaviors before students develop obesity. Universities could offer affordable gym memberships or discounts and counseling services, to support students in maintaining a healthy weight and active lifestyle.

In conclusion, in the setting of rising obesity prevalence, this study highlights the complex interplay of socio-demographic and lifestyle factors in the prevalence of obesity among university students in Germany. By identifying specific characteristics and the inherent heterogeneity within the student population, these findings highlight key areas for future research. Understanding the associations between student characteristics and obesity can aid in generating hypotheses and identifying high-risk subgroups that may benefit from tailored support.

## Supplementary Information

Below is the link to the electronic supplementary material.

Supplementary Material 1

## Data Availability

The data is private property of Constata GmbH. Constata GmbH has granted the right to publish this study. The data cannot be made publicly available. If you are interested in the data, please contact the Constata GmbH via: kontakt@constata.de . Via a contractual agreement the data can be shared confidentially with the Journal.
