# Supplementary material for: Obesity, food habits and socio-demographic factors among university students in Germany: a cross-sectional study
Source: Sci Rep. 2026 Jun 14;16:18374. doi: 10.1038/s41598-026-57347-y (PMC13265917; doi:10.1038/s41598-026-57347-y)
Supplement: Supplementary file 1 — Supplementary Material 1 [file 41598_2026_57347_MOESM1_ESM.docx]

Supplementary Material

**Table S1 Comparison Fachkraft 2020 and Sozialerhebung 2016**

|  |  | 10. Fachkraft Welle WS2016 | Sozial-erhebung 2016 |
| --- | --- | --- | --- |
| Share of Undergraduates | Enrolled in Bachelor program | 0,67 | 0,62 |
| Relationship status | unmarried relationship | 0,44 | 0,48 |
|  | married | 0,09 | 0,06 |
|  | no relationship | 0,46 | 0,46 |
| Migration background | At least one parent | 0,27 | 0,20 |
| Living situation | Parents/relatives | 0,23 | 0,20 |
|  | Individual housing or with partner | 0,28 | 0,38 |
|  | Student housing | 0,16 | 0,12 |
|  | Shared flat | 0,29 | 0,30 |
|  | Sub-renting | 0,04 | 0,01 |
| Parental Education | University degree or Fachhochschule (at least 1 parent) | 0,44 | 0,52 |
|  | University entrance degree (at least on parent) | 0,60 | 0,66 |
| Income | [€] | 958 | 918 |
| Number of universities |  | 477 | 371 |
| Student job |  | 0,67 | 0,68 |

Data from the Sozialerhebung 2016 ^23^ must not match Fachkraft data due to differences in sampling and weighting approaches. The presented variables are selected depending on further usage in the result section and availability in both datasets.

**Table S2 Descriptive statistics of used characteristics**

**Summary statistics: N mean sd min max median by(obesity: no obesity)**

|  | N | mean | sd | min | max | Median |
| --- | --- | --- | --- | --- | --- | --- |
| Age – [years] | 9337 | 23.22 | 3.41 | 18 | 40 | 23 |
| Gender [1=male, 2=female] | 9344 | 1.56 | .5 | 1 | 2 | 2 |
| UEL of 0 parents | 9364 | .39 | .49 | 0 | 1 | 0 |
| UEL of 1 parent | 9364 | .27 | .44 | 0 | 1 | 0 |
| UEL of 2 parents | 9364 | .34 | .47 | 0 | 1 | 0 |
| UD of 0 parents | 9364 | .55 | .5 | 0 | 1 | 1 |
| UD of 1 parent | 9364 | .25 | .43 | 0 | 1 | 0 |
| UD of 2 parents | 9364 | .2 | .4 | 0 | 1 | 0 |
| Migration status of parents | 7701 | .26 | .44 | 0 | 1 | 0 |
| Living situation - with parents or relatives | 9364 | .23 | .42 | 0 | 1 | 0 |
| Living situation - alone or with partner | 9364 | .27 | .44 | 0 | 1 | 0 |
| Living situation - student housing | 9364 | .16 | .37 | 0 | 1 | 0 |
| Living situation – shared flat | 9364 | .3 | .46 | 0 | 1 | 0 |
| Living situation - sub renting | 9364 | .04 | .2 | 0 | 1 | 0 |
| Relationship status – unmarried relationship, | 9364 | .45 | .5 | 0 | 1 | 0 |
| Relationship status – no relationship | 9364 | .46 | .5 | 0 | 1 | 0 |
| Relationship status - married | 9364 | .09 | .28 | 0 | 1 | 0 |
| Overall Income | 9363 | 958.06 | 925.46 | 0 | 15500 | 757 |
| Income - Public loan (Bafög) | 9363 | 103.24 | 214.11 | 0 | 909 | 0 |
| Income - Private loan | 9363 | 35.07 | 230.49 | 0 | 8600 | 0 |
| Income - private persons | 9363 | 353.92 | 628.88 | 0 | 12000 | 200 |
| Income – part-time employment | 9363 | 465.84 | 674.28 | 0 | 12000 | 260 |
| Preparation mode – self-cooking? | 9364 | 2.1 | .73 | 0 | 3 | 2 |
| Preparation mode – ready-made meals | 9364 | .93 | .68 | 0 | 3 | 1 |
| Preparation mode – canteens and restaurants | 9364 | 1.43 | .65 | 0 | 3 | 1 |
| Preparation mode – food delivery service | 9364 | .62 | .54 | 0 | 3 | 1 |
| Preparation mode – being cooked for | 9364 | 1.13 | .93 | 0 | 3 | 1 |
| Consumption frequency - fish | 9364 | .95 | .63 | 0 | 3 | 1 |
| Consumption frequency – meat | 9364 | 1.6 | .9 | 0 | 3 | 2 |
| Consumption frequency – vegetables | 9364 | 2.24 | .7 | 0 | 3 | 2 |
| Consumption frequency – cereals | 9364 | 2.02 | .66 | 0 | 3 | 2 |
| Consumption frequency – milk | 9364 | 2.16 | .83 | 0 | 3 | 2 |
| Consumption frequency – fruits | 9364 | 2.25 | .72 | 0 | 3 | 2 |
| Consumption frequency – savoury snacks | 9364 | 1.34 | .71 | 0 | 3 | 1 |
| Consumption frequency - sweets | 9364 | 1.65 | .78 | 0 | 3 | 2 |
| Age – [years] | 592 | 24.34 | 3.77 | 18 | 40 | 24 |
| Gender [1=male, 2=female] | 594 | 1.48 | .5 | 1 | 2 | 1 |
| UEL of 0 parents | 596 | .51 | .5 | 0 | 1 | 1 |
| UEL of 1 parent | 596 | .24 | .43 | 0 | 1 | 0 |
| UEL of 2 parents | 596 | .26 | .44 | 0 | 1 | 0 |
| UD of 0 parents | 596 | .65 | .48 | 0 | 1 | 1 |
| UD of 1 parent | 596 | .22 | .42 | 0 | 1 | 0 |
| UD of 2 parents | 596 | .13 | .34 | 0 | 1 | 0 |
| Migration status of parents | 488 | .35 | .48 | 0 | 1 | 0 |
| Living situation - with parents or relatives | 596 | .27 | .45 | 0 | 1 | 0 |
| Living situation - alone or with partner | 596 | .35 | .48 | 0 | 1 | 0 |
| Living situation - student housing | 596 | .15 | .36 | 0 | 1 | 0 |
| Living situation – shared flat | 596 | .19 | .39 | 0 | 1 | 0 |
| Living situation - sub renting | 596 | .04 | .2 | 0 | 1 | 0 |
| Relationship status – unmarried relationship, | 596 | .38 | .49 | 0 | 1 | 0 |
| Relationship status – no relationship | 596 | .49 | .5 | 0 | 1 | 0 |
| Relationship status - married | 596 | .12 | .33 | 0 | 1 | 0 |
| Overall Income | 596 | 953.06 | 940.29 | 0 | 9200 | 749.5 |
| Income - Public loan (Bafög) | 596 | 141.32 | 251.22 | 0 | 750 | 0 |
| Income - Private loan | 596 | 74.8 | 408.34 | 0 | 8000 | 0 |
| Income - private persons | 596 | 291.14 | 604.08 | 0 | 8700 | 100 |
| Income – part-time employment | 596 | 445.81 | 639.12 | 0 | 4850 | 227.5 |
| Preparation mode – self-cooking? | 596 | 2.03 | .77 | 0 | 3 | 2 |
| Preparation mode – ready-made meals | 596 | .98 | .7 | 0 | 3 | 1 |
| Preparation mode – canteens and restaurants | 596 | 1.38 | .67 | 0 | 3 | 1 |
| Preparation mode – food delivery service | 596 | .74 | .58 | 0 | 3 | 1 |
| Preparation mode – being cooked for | 596 | 1.13 | .99 | 0 | 3 | 1 |
| Consumption frequency - fish | 596 | 1.05 | .62 | 0 | 3 | 1 |
| Consumption frequency – meat | 596 | 1.86 | .83 | 0 | 3 | 2 |
| Consumption frequency – vegetables | 596 | 2.13 | .68 | 0 | 3 | 2 |
| Consumption frequency – cereals | 596 | 1.92 | .69 | 0 | 3 | 2 |
| Consumption frequency – milk | 596 | 2.09 | .82 | 0 | 3 | 2 |
| Consumption frequency – fruits | 596 | 2.06 | .74 | 0 | 3 | 2 |
| Consumption frequency – savoury snacks | 596 | 1.3 | .72 | 0 | 3 | 1 |
| Consumption frequency - sweets | 596 | 1.67 | .77 | 0 | 3 | 2 |
